# Is the evidence strong enough for acupuncture ameliorates clinical symptoms in patients with amyotrophic lateral sclerosis: A protocol for a systematic review and meta-analysis

**DOI:** 10.1097/MD.0000000000015218

**Published:** 2019-05-17

**Authors:** Qun Liao, Zunjiang Li, Hai Zeng, Xiaocong Feng, Wei Huang, Chuying Fu, Xiaolin Liang, Tian Li

**Affiliations:** The Second Clinical Medical College of Guangzhou University of Chinese Medicine, China.

**Keywords:** acupuncture, amyotrophic lateral sclerosis, meta-analysis, protocol, systematic review

## Abstract

**Background:**

Amyotrophic lateral sclerosis (ALS) is the most common form of motor neuron diseases. Until now, it lacks effective drugs for its treatment, and the median survival time of ALS is reported as only 20 to 48 months after the onset of symptoms. Acupuncture served as part of traditional Chinese therapy, has been widely applied to clinical practice for patients with ALS but lacks studies to verify its efficacy. This study provides a protocol of systematic review, with which we will comprehensively verify the effects of acupuncture on ALS with evidence-based studies.

**Methods:**

The eligible studies will be collected from 4 English databases (the MEDLINE via PubMed, the Cochrane Library, EMBASE, the Web of Science, and Ovid database), and 4 Chinese databases (China Science and Technology Journal Database, Chinese Biomedical Literature Database, Wan-fang Database, China National Knowledge Infrastructure) from October 2000 to October 2022. The primary outcome measure is the change in amyotrophic lateral sclerosis functional rating scale-revised (ALSFRS-R) scores. We will use RevMan V.5.3 software to calculate the data synthesis and will conduct meta-analysis based on the collected data.

**Results:**

The primary outcome measure is the change in ALSFRS-R scores, and secondary outcome measures included changes in forced vital capacity, grasping power, pinch strength, modified Norris Scale, ALS assessment questionnaire-40, and time to activity of daily living-independent will be measured and comprehensively assessed to evaluate the effect of acupuncture on ALS from this systematic review and meta-analysis.

**Conclusion:**

The systematic review and meta-analysis will assess the effect of acupuncture in the treatment of ALS with up-to-date clinical evidence.

**PROSPERO registration number:**

PROSPERO CRD 42019124785.

## 1 Introduction

Amyotrophic lateral sclerosis (ALS) is the most common form of motor neuron diseases (MND). It is characterized by degeneration of the upper and lower motor neurons, and is usually fatal within a few years of onset.^[1]^ In the clinic, it leads to severe limb and medullary disability and eventually results in respiratory failure,^[2]^ which is the main cause of death due to ALS^[3]^ with an accidence rate of 1.7 per 100,000.^[4]^ The median survival time of ALS is reported as 20 to 48 months after the onset of symptoms, among which 90% to 95% are sporadic ALS, and 5% to 10% of patients are familial ALS.^[5]^ The quality of life of patients with diagnosis as ALS is significantly impacted, these patients have severe and distressing symptoms accompanied by negative impacts in their physical, emotional, and social life, as they have to adapt to a rapidly debilitating ability of self-activity because of muscle weakness and eventual paralysis.^[6,7]^

Although many trials of ALS have been applied in clinic since the 1980s, Riluzole is the only drug of proven efficacy for treatment of ALS.^[8]^ Most ALS clinical trials actually lead to no substantial benefits, so the fact that many patients are often willing to experiment with unproven therapies.^[9]^ In a review by the Cochrane Library, it is reported that the probability of a 1-year survival improvement is only 9% with the application of Riluzole.^[10]^ Besides, Riluzole is so expensive that many patients with ALS cannot support treatment costs. The most common adverse effects of Riluzole are abdominal pain, constipation, and elevated alanine aminotransferase. In 2017, Edaravone was accepted as the second drug approved in the treatment for ALS patients.^[11]^ Edaravone can reduce the oxidative stress in the progress of ALS, but its exact mechanism is still unknown, and the role of Edaravone in delaying the progression of ALS in some patients only has slight effects in some trials, thus highly safe and effective therapies are still in need for the treatment of ALS.

ALS is a progressive and fatal neurodegenerative disease. The pathogenesis of this disease is still unclear. There is no effective method for western medicine treatment. At present, there is an increasing demand for supplementary and alternative therapies applied in improving the symptoms and delaying the progress of ALS.^[7–9]^ Traditional Chinese medicine acupuncture, as part of supplemental and alternative therapies, has a significant effect on the treatment of ALS. It could improve clinical symptoms such as the muscle weakness and ameliorate the quality of life of patients.^[10,11]^ Acupuncture has a history of more than 2100 years in China. It is an alternative treatment that is incorporated into the US health insurance program in 2018 and is increasingly recognized by global public and health management professionals.^[12]^ Through clinical observation, acupuncture can affect multiple systems of the body, including the nervous system, immune system, cardiovascular system, and so on.^[13,14]^ Some clinical studies have also shown that acupuncture can improve the physical activity of ALS patients and improve the muscle strength of the limbs. Acupuncture can also eliminate the severity of clinical symptoms and restore the ability for normal activity in their daily life.^[15]^ Acupuncture effectively improve muscle atrophy, muscle weakness, speech dysphagia, and other symptoms in patients with ALS as well.^[16]^ In China, it is applied in clinical trials in some hospital and results in better outcomes by comparing with control groups.

However, according to present literature, there is still no systematic review of the randomized controlled trials (RCTs) of acupuncture for the efficacy and safety of ALS. Therefore, this review will comprehensively analyze the effects of acupuncture on ALS. The current systematic review aims to critically assess whether acupuncture is an effective and safe treatment for ALS according to the literatures published from 2000 to 2022.

## 2 Method

### 2.1 Inclusion criteria for study selection according to participants, interventions, comparison, outcomes, study designs criteria

#### 2.1.1 Participants

All patients age 18 to 85 years old diagnosed with the El Escorial diagnostic criteria of the World Federation of Neurology.^[17]^ The pregnant women will be excluded. The studies that the relevant mean and standard deviation of the clinic indication cannot be extracted from the original literature or by contacting the corresponding authors will not be included. All gender or race will be included. Any patient with surgery or serious complications caused by the other diseases (hypertensive heart disease, diabetes, rheumatic myopathy, renal failure, etc) will be excluded. Patients with ALS caused by non-MND diseases will be excluded as well.

#### 2.1.2 Interventions and comparison

In eligible researches, the control group was treated with conventional western medicine (eg, Riluzole, Edaravone, etc), and the intervention of the experimental group regarded acupuncture for the treatment of ALS alone or based on the conventional western medicine.

#### 2.1.3 Outcomes measures

The primary outcome measure is the change in ALS functional rating scale-revised scores compared with baseline. Secondary outcome measures included changes in forced vital capacity, grasping power, pinch strength, modified Norris Scale, ALS assessment questionnaire-40, and time to activity of daily living-independent. Adverse events will also be assessed by comparing with different groups.

#### 2.1.4 Study design

We will include RCTs of acupuncture therapy for ALS that have control groups (placebo control group or conventional drug treatment control group). All studies describe specific randomized grouping methods or simply mention randomization in the study, such as computer software to generate random numbers or random number tables. No language, time, geographical area, sample size, or publication status restriction.

### 2.2 Data search method

**Figure 1:**
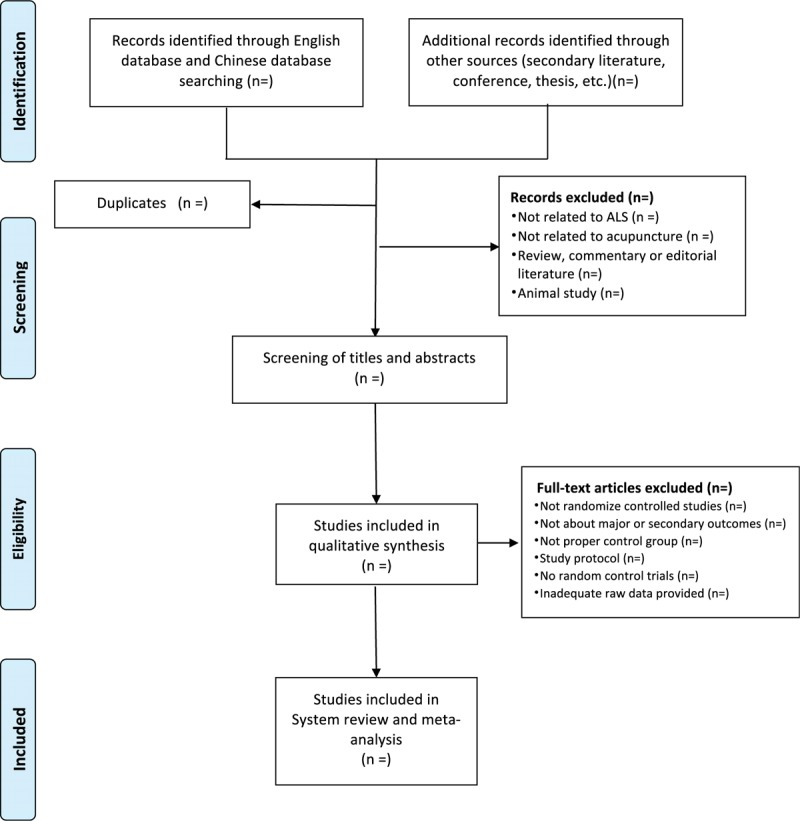
PRISMA flow chart of study selection process in the systematic review. PRISMA = preferred reporting items for systematic review and meta-analysis.

All the trials will be searched according to inclusion criteria through 4 English databases and 4 Chinese databases from October 2000 to October 2022. The 4 English databases are the MEDLINE via PubMed, the Cochrane Library, EMBASE, and the Web of Science, and 4 Chinese databases are the China Science and Technology Journal Database, Chinese Biomedical Literature Database, Wan-fang Database, and China National Knowledge Infrastructure. Standards-compliant conference articles will be removed. Studies removed after full-text review will be recorded with specific exclusion reason. Any selected study will fulfill the inclusion criteria. The search topics will be composed of P + I + C +O + S, P + I + C + O, and P + I + O in order to ensure the highest search rate of literature search. The preferred reporting items for systematic review and meta-analysis flow diagram shows the selection process of eligible papers^[17,18]^ (Fig. 1).

### 2.3 Risk of bias assessment

The Cochrane Risk of Bias tool will be used to assess bias of the included studies according the following items (random sequence generation and allocation concealment [selection bias]; binding of participants and personnel [performance bias]; binding of outcome assessment [detection bias]; incomplete outcome data [attrition bias]; selective reporting [reporting bias]; and other potential sources of bias). While the Collaboration Review Manager software (Version 5.3) will be used to evaluate their methodological quality. All the items will be evaluated by 2 independent reviewers, and any divergence will be resolved by discussion or the third reviewer as well.

### 2.4 Data extraction and management

#### 2.4.1 Data extraction

The same 2 reviewers will extract published year, first author name, average age of patients, sample size, duration, details of the treatment and control group, details of the outcomes, and adverse events. An arbiter for a final decision throughout the entire procedure will access the implementation process.

#### 2.4.2 Quantitative synthesis

We will compare the change from baseline to endpoint of the indications for continuous data. If mean difference standard error (SD_change_) were not provided, we would calculate the SD_changed_ with the following formula. We would only choose the last available time point reported on the indications if the study had different time points throughout the intervention.  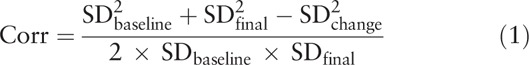



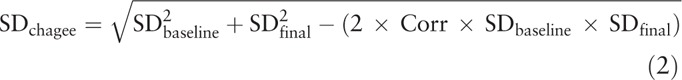


#### 2.4.3 Selection of effects model

The standard mean difference with 95% confidence interval (CI) will be used to evaluate the continuous data, while dichotomous outcomes will be measured with the rate ratio (RR) with 95% CIs. *I*^2^ will be used to assess heterogeneity of the studies. If *I*^2^ < 50, we would perform the fixed-effects model to calculate the RR and mean difference, while we would choose the random-effects model if *I*^2^ ≥ 50.

### 2.5 Analysis of the data

#### 2.5.1 Assessment of heterogeneity

The chi-squared test will be applied to evaluate the heterogeneity with the cut off value of *I*^2^ according to the guideline of the Cochrane Handbook. If *I*^2^ >50%, trials would be considered with significant heterogeneity, and subgroup analysis will be necessarily performed to assess the potential heterogeneity sources.

#### 2.5.2 Subgroup analysis

If it was considered with high heterogeneity, subgroup analysis will be performed to investigate whether the onset time, duration of illness, age, number of samples, and so on to cause the heterogeneity, which will ensure the credibility of our results.

#### 2.5.3 Sensitivity analysis

We will also apply sensitivity analysis to evaluate the robustness and reliability of Methodological quality, heterogeneity, studies quality (according to the 4 levers: high, moderate, low, or very low), and sample characteristic of included studies.

#### 2.5.4 Assessment of reporting biases

We will conduct analysis of Egger publication bias plot and Begg funnel plot with pseudo 95% confidence limits to determine the publication bias in all the literature with sufficient studies.

#### 2.5.5 Test sequential experiment

In order to illustrate and confirm the credibility of our results, we performed a sample size analysis by trial sequential analysis, which aims to rule out the possibility of false positives.

### 2.6 Ethics approval

Our data will be extracted from the published studies through database, and there is not directly related to patients’ data, no ethical approval is required. The findings of this systematic review will provide implication of the effectiveness of acupuncture for ALS. The systematic review will be disseminated in a peer-reviewed journal and published at conference presentations, which will be served as the guidance for a multi-center random control trial in our further research.

## 3 Discussion

ALS is a progressive and fatal neurodegenerative disease. Most patients survive only 3 to 5 years after diagnosis, and eventually die of respiratory failure,^[1,4]^ but the pathogenesis of this disease is still unknown. There is no effective method for Western medicine treatment, thus ALS currently lacks effective therapeutic drugs. Now the FDA-approved Riluzole and Edaravone are only limited to short-term prolonged patients’ survival,^[8,18]^ without fundamentally relieving the symptoms of ALS. And their prices are relatively expensive, so many patients cannot afford it if a long duration was in need.

In China, the application of traditional Chinese therapy has a long-term experience and there is a wide indication that traditional Chinese therapy could be used in the treatment of ALS without little side effects.^[14–16]^ In recent decades, both experimental researches and clinical trials have showen that acupuncture, as part of supplemental and alternative therapies, has a significant effect on the neurological diseases such as ALS and are gradually being used in clinical practice.^[19,20]^ According to China's current national conditions, such a populous country and developing country, finding effective and low-toxic methods for treating ALS patients from traditional Chinese therapy, and even developing new therapeutic methods, is undoubtedly the gospel for all patients with ALS. Therefore, the effect of acupuncture on ALS is worth exploring through our reviews based on the published studies from 2000 to 2022. However, there are limitations in this systematic review that may affect the drawn conclusion. First, the included trials are mainly restricted to the published results, which may run risk of publishing bias. Second, different times of onset and types of ALS may lead to heterogeneity in our results in terms of that subgroup analysis may be unavailable if the quantities of included studies are not enough. Thus, in order to avoid these, we will conduct this analysis with more studies and more extra unpublished results as possible.

## Author contributions

Tian Li designed the study and made all the decisions when came across disagreement during the analysis. Zunjiang Li and Qun Liao were responsible for data search, extraction, and writing. Wei Huang, Xiaocong Feng, and Hai Zeng performed data analysis and evaluated the accuracy of the whole process. Hai Zeng, Chuying Fu, and Xiaolin Liang provided support for modifications of English writing. All authors contributed to the evaluation of review and analysis and approved the final version submitted for publication

**Conceptualization:** Qun Liao, Chuying Fu, Xiaolin Liang, Tian Li.

**Data curation:** Zunjiang Li, Hai Zeng, Xiaocong Feng, Chuying Fu, Tian Li.

**Formal analysis:** Qun Liao, Zunjiang Li.

**Methodology:** Qun Liao, Zunjiang Li, Hai Zeng, Xiaocong Feng, Wei Huang, Tian Li.

**Software:** Hai Zeng, Chuying Fu.

**Supervision:** Xiaocong Feng.

**Validation:** Xiaolin Liang.

**Writing – original draft:** Zunjiang Li, Wei Huang, Tian Li.

**Writing – review and editing:** Qun Liao, Xiaolin Liang, Tian Li.

Tian Li orcid: 0000-0003-2342-5645.

## References

[1] Pratt AJ Getzoff ED Perry JJ Amyotrophic lateral sclerosis: update and new developments. Degener Neurol Neuromuscul Dis 2012;2012:1–4. 23019386 10.2147/DNND.S19803 PMC3457793

[2] Hardiman O Al-Chalabi A Chio A Amyotrophic lateral sclerosis. Nat Rev Dis Primers 2017;3:17071. 28980624 10.1038/nrdp.2017.71

[3] Christidi F Karavasilis E Rentzos M Clinical and radiological markers of extra-motor deficits in amyotrophic lateral sclerosis. Front Neurol 2018;9:1005. 30524366 10.3389/fneur.2018.01005 PMC6262087

[4] Oskarsson B Gendron TF Staff NP Amyotrophic lateral sclerosis: an update for 2018. Mayo Clin Proc 2018;93:1617–28. 30401437 10.1016/j.mayocp.2018.04.007

[5] Luo L Song Z Li X Efficacy and safety of edaravone in treatment of amyotrophic lateral sclerosis-a systematic review and meta-analysis. Neurol Sci 2018;40:235–41. 30483992 10.1007/s10072-018-3653-2

[6] Oberstadt M Esser P Classen J Alleviation of psychological distress and the improvement of quality of life in patients with amyotrophic lateral sclerosis: adaptation of a short-term psychotherapeutic intervention. Front Neurol 2018;9:231. 29713302 10.3389/fneur.2018.00231 PMC5911468

[7] Valadi N Evaluation and management of amyotrophic lateral sclerosis. Prim Care 2015;42:177–87. 25979579 10.1016/j.pop.2015.01.009

[8] Zoccolella S Beghi E Palagano G Riluzole and amyotrophic lateral sclerosis survival: a population-based study in southern Italy. Eur J Neurol 2007;14:262–8. 17355545 10.1111/j.1468-1331.2006.01575.x

[9] Kiernan MC Vucic S Cheah BC Amyotrophic lateral sclerosis. Lancet 2011;377:942–55. 21296405 10.1016/S0140-6736(10)61156-7

[10] McGown A Stopford MJ High-throughput drug screens for amyotrophic lateral sclerosis drug discovery. Expert Opin Drug Discov 2018;13:1015–25. 30317895 10.1080/17460441.2018.1533953

[11] Schultz J Disease-modifying treatment of amyotrophic lateral sclerosis. Am J Manag Care 2018;24 15 Suppl:S327–35. 30207671

[12] The United States Federal Acupuncture Insurance Act won the first battle and was approved by the House Committee Fundraising Committee. Available at: https://mp.weixin.qq.com/s/ujOzzBgC3yR86zRFfNFPNAhl=1; 2018.

[13] Hu B Bai F Xiong L The endocannabinoid system, a novel and key participant in acupuncture's multiple beneficial effects. Neurosci Biobehav Rev 2017;77:340–57. 28412017 10.1016/j.neubiorev.2017.04.006

[14] Liu X Li A Hu J Clinical research status of acupuncture and moxibustion in the treatment of amyotrophic lateral sclerosis. Asia-Pac Tradit Med 2017;13:73–5.

[15] Meng B Tian J Clinical observation of Tong Du Wen Yang needling for amyotrophic lateral sclerosis. Shanghai J Acupunct Moxibust 2017;36:134–7.

[16] Chen X Zhang G Zhang M Clinical research of integrated traditional Chinese and Western medicine in the treatment of amyotrophic lateral sclerosis. J Chin Med 2012;27:1214–5.

[17] Nozari A Bagchi A Saxena R Neuromuscular Disorders and Other Genetic Disorders. Miller's Anesthesia, Eighth Edition; 2015:1266–1286.

[18] Rothstein JD Edaravone: a new drug approved for ALS. Cell 2017;171:725. 29100067 10.1016/j.cell.2017.10.011

[19] Bedlack RS Joyce N Carter GT Complementary and alternative therapies in amyotrophic lateral sclerosis. Neurol Clin 2015;33:909–36. 26515629 10.1016/j.ncl.2015.07.008 PMC4712627

[20] Sudhakaran P Amyotrophic lateral sclerosis: an acupuncture approach. Med Acupunct 2017;29:260–8. 29067136 10.1089/acu.2017.1241 PMC5653341

